# Isomin: a novel cytoplasmic intermediate filament protein from an arthropod species

**DOI:** 10.1186/1741-7007-9-17

**Published:** 2011-02-28

**Authors:** Caterina Mencarelli, Silvia Ciolfi, Daniela Caroti, Pietro Lupetti, Romano Dallai

**Affiliations:** 1 Department of Evolutionary Biology, University of Siena, Via Aldo Moro 2, 53100 Siena, Italy

## Abstract

**Background:**

The expression of intermediate filaments (IFs) is a hallmark feature of metazoan cells. IFs play a central role in cell organization and function, acting mainly as structural stress-absorbing elements. There is growing evidence to suggest that these cytoskeletal elements are also involved in the integration of signalling networks. According to their fundamental functions, IFs show a widespread phylogenetic expression, from simple diblastic animals up to mammals, and their constituent proteins share the same molecular organization in all species so far analysed. Arthropods represent a major exception in this scenario. Only lamins, the nuclear IF proteins, have so far been identified in the model organisms analysed; on this basis, it has been considered that arthropods do not express cytoplasmic IFs.

**Results:**

Here, we report the first evidence for the expression of a cytoplasmic IF protein in an arthropod - the basal hexapod *Isotomurus maculatus*. This new protein, we named it isomin, is a component of the intestinal terminal web and shares with IFs typical biochemical properties, molecular features and reassembly capability. Sequence analysis indicates that isomin is mostly related to the Intermediate Filament protein C (IFC) subfamily of *Caenorhabditis elegans *IF proteins, which are molecular constituents of the nematode intestinal terminal web. This finding is coherent with, and provides further support to, the most recent phylogenetic views of arthropod ancestry. Interestingly, the coil 1a domain of isomin appears to have been influenced by a substantial molecular drift and only the aminoterminal part of this domain, containing the so-called helix initiation motif, has been conserved.

**Conclusions:**

Our results set a new basis for the analysis of IF protein evolution during arthropod phylogeny. In the light of this new information, the statement that the arthropod phylum lacks cytoplasmic IFs is no longer tenable.

See commentary article: http://www.biomedcentral.com/1741-7007-9-16.

## Background

Intermediate filaments (IFs) are major cytoskeletal elements of metazoan cells. They form an integrated system that extends from the cell membrane to the nucleus and, by anchoring at intercellular junctions, contribute to coordinate individual cells into tissues (reviewed in [1,2]). The mechanical properties of IFs are crucial for the maintenance of cell shape and tissue integrity, both in the adult organism and during embryonic development and differentiation of specific tissues. Being very strong and extensible elements, they provide the cell with unique mechanical properties and act as stress-absorbing cytoskeletal components. Recently, it has been proposed that IFs act as a scaffold for the transduction of not only mechanical perturbations but also of other types of signals from the exterior to all internal compartments of the cell and, from this, the idea of IFs as 'regulatory platforms' implicated in the regulation of key signalling pathways has emerged [2]. IF proteins are encoded by a large family of genes, which includes both nuclear lamins and cytoplasmic IF proteins; their expression is developmentally-regulated and tissue-specific [1]. Consistent with the central role of IFs in cell function, mutations in genes encoding IFs have been shown to cause, or predispose, to more than 30 different human diseases [3].

All members of the IF family share a typical tripartite molecular organization which has been conserved during metazoan evolution. It consists of a central assembly-competent α-helical domain with coiled-coil forming ability - the so-called rod domain - and of amino and carboxyterminal domains, named the head and the tail domain, respectively, which are variable in length, sequence and properties [4]. The rod domain is divided into subdomains (coils 1a, 1b, 2a and 2b) by short non-helical linkers (L1, L12, L2). The length of the rod and of its different subdomains is defined and conserved across species. Nuclear lamins are characterized by a longer rod domain, due to an extra 42 residues in their coil 1b subdomain [4,5]. For cytoplasmic IF proteins, phylogenetic surveys have shown evidence of the occurrence of two molecular prototypes, which segregate according to phylogenetic lineages: the L-type, which shares with lamins a longer rod domain and is expressed in protostome phyla; and the S-type, endowed with a shorter rod domain, which is thought to be arisen from the L-type by a deletion event and, until now, has only been detected in the three deuterostome chordate phyla [6]. On this basis, it has been speculated that cytoplasmic IFs arose early in evolution from a mutated lamin gene [7]. Duplications of IF genes followed by diversification and specialization of the new genes have occurred during the evolution of most phyla. The complexity of the cytoplasmic IF protein repertoire expressed in different metazoan phyla thus varies, reaching its maximum in vertebrates which express up to 70 proteins belonging to six distinct IF subfamilies [8].

In such a scenario, arthropods, the largest metazoan phylum, are a major exception. In fact, both electron microscopy [9] and molecular cloning studies [6,10] have been unable to detect any cytoplasmic IF protein in these organisms, although they do express a nuclear IF system made of authentic lamins. Other cytoskeletal components have been proposed to have assumed, in arthropods, the mechanical functions that are usually played by cytoplasmic IFs in other organisms (discussed in [11]). For example, wing epithelial cells are stabilized in insects by a cytoskeletal array consisting of parallel bundles of 15-protofilament microtubules and actin filaments [12]. However, the absence of cytoplasmic IFs in this phylum is still puzzling.

In the collembolan species belonging to the genera *Isotomurus *and *Isotoma *the organization of the midgut epithelium is unusual for the presence of a peculiar, filamentous web [13,14] which has been preliminarily shown not to consist of actin, contrary to what is seen in the vertebrate intestinal terminal web [15]. We describe the identification and molecular characterization of the protein forming such a peculiar cytoskeletal array. This protein, that we named isomin, shares with cytoplasmic IFs typical molecular features and *in vitro *assembly properties. Thus, our results provide the first evidence of a cytoplasmic IF protein expressed in an arthropod species.

## Results

### A peculiar terminal web in Isotomurus midgut epithelium

**Figure 1:**
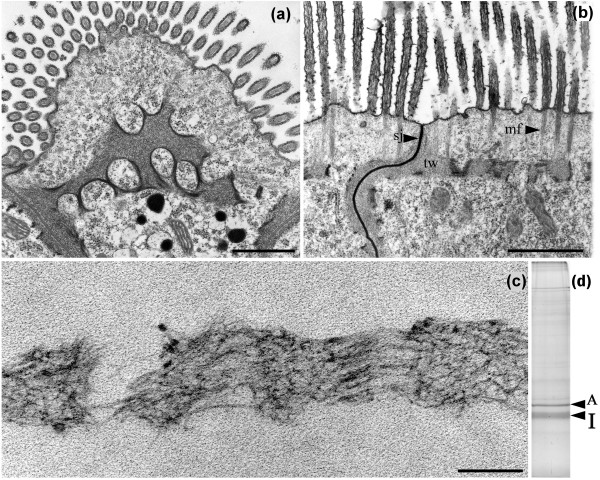
**Electron microscopy and electrophoretic analysis of *Isotomurus *intestinal terminal web**. (a,b) The terminal web (tw) is a discontinuous structure formed by filaments which contact the septate junction (sj). Microfilaments (mf) descending from microvilli insert onto the terminal web. Bars = 1 μm. (c) Electron microscopy analysis of cytoskeletal Triton-resistant midgut preparations obtained as described in Material and methods: the terminal web is not affected by treatment with detergent and high ionic strength solutions. Bar = 200 nm. (d) Electrophoretic analysis on an 8% SDS-polyacrylamide gel of the detergent-insoluble fraction from *Isotomurus *intestine: a major protein band (I) is present, which migrates immediately below actin (A).

Collembolan species belonging to the genus *Isotomurus *express, in their midgut epithelium, a peculiar terminal web- consisting of a dense belt-like layer of closely intertwined filaments of about 8-10 nm in diameter, which crosses the apical cytoplasm and contacts laterally the membrane at the septate junction level where filaments adhere to and reinforce the cytoplasmic face of the junction (Figure 1a and 1b). This cytoskeletal array appears to act as an anchoring structure for the microfilament bundles descending from microvilli and segregates the cytoplasm into an apical and a basal region that are structurally distinct, the latter containing most part of the cellular organelles. The web is not continuous but shows several fenestrations which appear to be somehow reinforced at their edges (Figure 1a and 1b).

We have preliminarily reported that the web does not consist of actin, since it is not decorated by specific antibodies or by heavy meromyosin fragments and does not disassemble after treatment with cytochalasin B [15]. Also, it is strongly resistant to extraction with high salt/detergent-containing solutions, which solubilize most cytoplasmic components (Figure 1c). The main component of the residual fraction is a protein with an apparent molecular weight of about 40 kDa which migrates immediately below actin after SDS-page (Figure 1d).

The ultrastructural organization of the apical epithelial region in the midgut of *Isotomurus *sp. is strongly reminiscent of the terminal web described in the intestine of some nematode species [16]. In *Caenorhabditis elegans*, the web contains a group of IF proteins [17]. The possibility that the *Isotomurus *filamentous web is also assembled by IF-related proteins is, indeed, suggested by its insolubility properties. On this basis, and in order to confirm this hypothesis, we decided to characterize the 40 kDa protein (that we named isomin). at the molecular level and to ascertain its relationship with the intestinal web.

### Sequencing and molecular analysis of isomin protein

Isomin was first enzymatically digested and the major tryptic peptides were analysed by mass spectrometry. This analysis provided a series of short aminoacid sequences that did not match with any protein in protein or Expressed Sequence Tags (EST) databases. However, some of these fragments contain a definite stretch of aminoacids that allowed the design of degenerate sense and antisense primers (Additional File 1, Table S1). Among the primer combinations we tested, only one (B2/D1) was successful. A series of reverse-transcriptase polymerase chain reaction (RT-PCR) experiments resulted in the amplification of a 360 bp fragment, which was cloned and sequenced on both strands. The corresponding deduced amino acid sequence (120aa) contains some of the partial aminoacid sequences obtained by mass spectrometry, thus confirming the specificity of the amplified sequence (Additional File 2, Figure S1). The complete isomin sequence was successively obtained by 5'-3' rapid amplification of  complementary DNA ends (RACE) using specific primers designed on the 360 bp sequence. The full-length complementary DNA is 1570 bp: it contains a 1236 bp open reading frame corresponding to a deduced amino acid sequence of 411 residues, with a predicted molecular mass of 48,036 Da and a theoretic isoelectric point of 6.65. The open reading frame is flanked by 5' and 3' untranslated regions of 72 bp and 262 bp, respectively, with a polyadenylation signal located 15 bp upstream of the polyA tail (Additional File 2, Figure S1).

A BLASTp search for isomin homologues resulted in the 10 best scores reported in Table 1. Among these, eight protein sequences are deduced from the genome of different *Drosophila *species and are annotated in Flybase as potential members of the Pfam family of intermediate filament proteins: the presence of the nuclear localization signal suggests that these proteins are lamins. The remaining two sequences are IF proteins from *Danio rerio *and *Ciona intestinalis*. The observed percentage of similarity between the identified proteins and isomin (24%-25% identity, 40%-43% positives) is in the range usually found among IF proteins, which primarily conserve sequence principles rather than actual sequences.

**Table 1:** BLASTP best 10 alignments.

**Accession No**.	Species	Description	% Identity	E-value
XP_001985993.1	*Drosophila grimshawi*	Gene product from transcript GH21120-RA *	25%	4e-06

XP_002049707.1	*D. virilis*	Gene product from transcript GJ21743-RA *	25%	3e-04

XP_002016166.1	*D. persimilis*	Gene product from transcript GL10641-RA *	24%	3e-04

XP_001360601.1	*D. pseudoobscura*	Gene product from transcript GA10086-RA *	24%	3e-04

XP_002063755.1	*D. willistoni*	Gene product from transcript GK15736-RA *	25%	0.002

XP_001002383.1	*Danio rerio*	Hypothetical protein LOC436656 ^†^	25%	0.005

XP_001975619.1	*D. erecta*	Gene product from transcript GG22418-RA *	24%	0.010

XP_002091326.1	*D. yakuba*	Lamin C	24%	0.010

XP_001988956.1	*D. grimshawi*	Gene product from transcript GH10295-RA *	24%	0.022

XP_001027681.1	*Ciona intestinalis*	Intermediate filament protein IF-A	24%	0.026

* All the *Drosophila *genes are classified by Pfam as intermediate filament proteins and are homologs of lamin sequences.

^† ^This gene has been characterized as cytokeratin E7.

**Figure 2:**
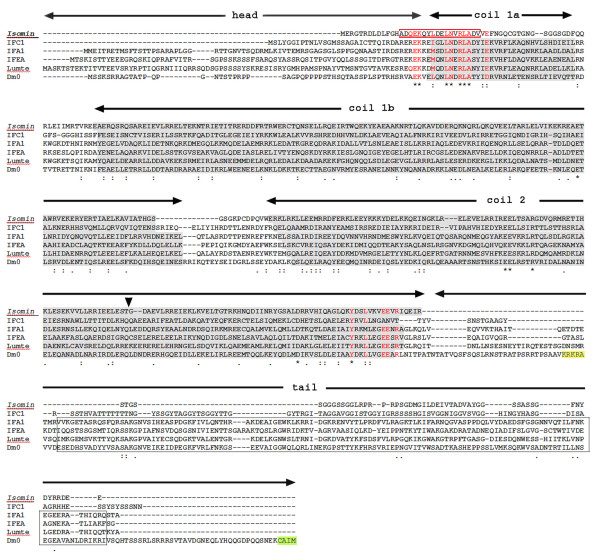
**Alignment of the isomin sequence with other intermediate filaments (IFs) reveals that isomin shares the IF tripartite molecular organization**. Sequences reported in figure are representatives of protostome cytoplasmic IFs and lamins. They include: two *Caenorhabditis elegans *proteins, IFC-1 [Swiss-Prot: O45168] and IFA-1 [Swiss-Prot: P90901]; the non-neuronal IF protein from the mollusc *Helix aspersa *(IFEA, [Swiss-Prot:P22488]); the IF protein from the annelid *Lumbricus terrestris *(Lumte, [Swiss-Prot: Q25421]), and lamin Dm0 from *Drosophila melanogaster *[Swiss-Prot: P08928]. Predicted coiled coil segments forming the central rod domain are shaded in grey. The rod domain is delimited by the conserved helix initiation and helix termination motifs; in the figure, those residues of these signature sequences that are shared by both isomin and at least one of the other IF sequences are evidenced in red. The different coiled coil subdomains within the rod are indicated by horizontal lines. The isomin region still endowed with coiled coil-forming capability that contains most of the helix initiation motif and partly coincides with the beginning of coil 1a in other IFs is boxed in red. The discontinuity in isomin coil 2 that occurs in correspondence of the stutter region of other IFs is marked by an arrowhead. In the lamin sequence, the nuclear signature motif and the terminal CaaX box are evidenced in yellow and in green, respectively; the lamin homology domain, occurring in all the sequences reported in the figure except isomin and IFC-1, is boxed in grey. Symbols underlying the sequence alignment indicate aminoacids that at a given position are identical (*), strongly similar (:) or weakly similar (.).

According to the BLAST results, a secondary structure prediction analysis indicates that isomin possesses a molecular organization closely resembling the typical tripartite structure of IF proteins, with non-helical head and tail domains encompassing a central coiled coil rod which is, in turn, divided in subdomains. Figure 2 documents such a similarity by aligning isomin sequence and the predicted domain organization with those exhibited by *Drosophila *lamin C and by a series of proteins representative of protostome cytoplasmic IFs (see also Additional File 3, Figure S2 for the heptad repeat along the three coiled coil domains)

As in all IF proteins, the isomin rod domain is delimited by conserved aminoacid sequences, the so-called helix initiation and helix termination motifs [4] and is divided into three distinct coiled-coil subdomains by short non-helical linker segments (Figure 2). The consensus sequence at the beginning of the isomin rod shows the absolutely conserved sequence LNXR. Within the helix termination motif, the consensus sequence of the so-called IFA epitope (YRKLLEGEE) appears to be poorly conserved. However, the reactivity with the IFA (intermediate filament antigen) monoclonal antibody is much less conserved among invertebrate phyla than it is in vertebrates [9,18] and the occurrence of IF proteins which do not contain the canonical IFA sequence has been reported [19].

Apart from very short variations, isomin coil 1b and coil 2 display the conserved length observed in other IF proteins. In particular, coil 1b exhibits the longer prototype typical of lamins and of protostome cytoplasmic IFs [6]. As in other invertebrate IF proteins (for example [20]), coil 2 is a continuous coiled coil domain, which is not predicted to be interrupted by a non-helical linker region in the two subdomains usually indicated as coil 2a and coil 2b in the consensus IF structure. However, crystallographic data have also recently indicated a continuous coiled coil structure for this domain in vertebrate vimentin [21]. In all IFs, an obligatory feature is the presence of a stutter - a discontinuity in the heptad repeat pattern that is equivalent to an insertion of four extra residues at the end of a heptad. The position of the stutter is quite conserved, indicative of its fundamental role in filament assembly [4]. In isomin, coil 2 also exhibits at this position a discontinuity in the heptade phasing. It should be noted that such a discontinuity corresponds to the insertion of two (f, g) - rather than four (d, e, f, g) - extra residues (Figure 2; Additional File 3, Figure S2). Interestingly, the alignment of isomin with other IF proteins reveals a gap of two residues in the protein just at this site (Figure 2). The same feature is also present in the IFC-2 protein from *C. elegans *[20]. Thus, isomin shares the consensus organization of coil 1b and coil 2 segments in IF proteins but, on the contrary, coil 1a shows peculiar features. This region can be easily identified by the occurrence of the helix initiation motif. However, the subsequent aminoacid sequence is profoundly altered, the heptade repeat underlying the coiled-coil structure is lost and only a residual coiled coil-forming capability is predicted at residues 15-33, which contain the conserved helix initiation motif (Figure 2; Additional File 3, Figure S2). A brief sequence rich in glycine and serine residues follows the conserved motif, forming a region of high predicted flexibility (data not shown). This is a structural feature that, in most IF proteins, characterizes the L1 spacer and which is crucial for the correct IF assembly [4].

Besides the hydrophobic interactions involving the apolar residues of the heptad repeats, the correct assembly of IFs is specified and stabilized by the formation of both intra- and interchain salt bridges arising from the regular disposition of oppositely charged residues [4]. Like other IF proteins, isomin contains a high percentage of charged residues. The observed linear distribution of acidic and basic residues within coil 1b and coil 2 could allow the formation of several stabilizing ionic interactions. Both coil 1b and coil 2 are basic segments, with an isoelectric point of 8.78 and 9.39, respectively. This is quite an unusual feature among IF proteins which, most commonly, possess acidic coiled-coil segments. Basic coiled coil domains, however, also occur in a few IF proteins from *C. elegans *(coil 1a of the IFA-3 protein, coil 1b in IFD-1 and in IFD-2).

No obvious sequence relationship is evident with other IF proteins along the isomin head and tail regions (Figure 2). Both the head and the tail are short acidic segments. The tail domain lacks the hallmark motifs of nuclear lamins (the nuclear localization signal and the C-terminal CaaX box) thus confirming that isomin is a cytoplasmic IF protein. It shows a peculiar internal stretch of alternating arginine and proline (residues 371-376) and a high content in glycine (20.4%) and serine (18.5%). The latter feature relates the isomin tail domain to the unusual tail of *C. elegans *IFC-1 and IFC-2 proteins [20]. Similarly, the isomin tail lacks the lamin-homology domain that is present in the tail domain of most protostome IF proteins except a few proteins, among which are *C. elegans *IFC-1 and IFC-2 proteins [6,20].

IF proteins can undergo multiple posttranslational modifications. In particular, phosphorylation is crucial for the control of IF dynamics and function [22]. *In vivo*, this posttranslational modification is modulated by many kinases and phosphorylated sites are clustered in the accessible head and/or tail domains, probably because of limited kinase access along the coiled coil rod domain [22]. Like other IFs, isomin contains several sites that are predicted to be specifically phosphorylable by different kinases (Additional File 4. Figure S3); these are more abundant in the tail domain (14.8% of the domain aminoacid content) than in the rod (6.4%) or in the very short head domain, which contains only one predicted phosphorylable threonine residue.

Two sumoylation sites are also predicted at Lys^308 ^and Lys^347 ^(Additional File 4, Figure S3). This protein modification - which has been recently reported to occur also on cytoplasmic intermediate filaments expressed in the hemidesmosome-like structures of *C. elegans *epidermis - has been implicated in the regulation of IF assembly, affecting the exchange rate between the soluble and the polymerized IF fraction [23].

### Isomin is a true component of the *Isotomurus *terminal web

**Figure 3:**
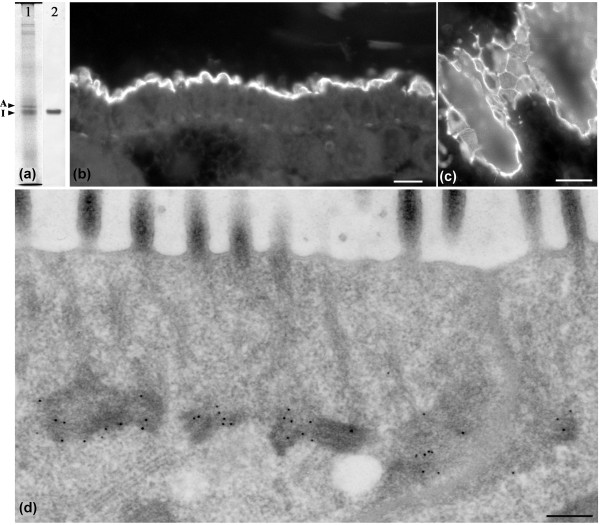
**Isomin is a constituent of *Isotomurus *terminal web**. (a) Specificity of the anti-isomin polyclonal antibodies: (1) Coomassie-blue staining; (2) immunostaining. A = actin; I = isomin. Electrophoresis on an 8% SDS-polyacrylamide gel. (b, c) Immunofluorescence staining of paraffin-embedded intestine sections. Bars = 20 μm. (d) Immunoelectron microscopy localization of isomin at the terminal web. Bar = 200 nm.

In order to confirm that isomin is a true component of the *Isotomurus *terminal web, specific polyclonal antibodies were raised against the recombinant 120 aa protein fragment, obtained by cloning of the 360 bp amplification product (Additional File 2, Figure S1): the specificity of the affinity-purified antibodies is shown in Figure 3a. Immunofluorescence experiments on *Isotomurus *whole-body sections revealed a strong specificity of the staining for the midgut epithelium, since no other tissue reacted (data not shown). Only the apical region of the midgut epithelium was strongly stained (Figure 3b). Sections tangential to the epithelial surface showed a reticulate staining within the cell, which is compatible with the occurrence of a subapical network of filaments, and a reinforcement of the fluorescent signal along cell boundaries where the filaments of the web adhere to and run along the intercellular junctions (Figure 3c).

Localization of isomin by immunoelectron microscopy experiments unequivocally established that this protein is a specific component of the *Isotomurus *midgut terminal web. Gold labelling was localized exclusively on the filamentous web and no other cellular components were labelled by the anti-isomin antibodies (Figure 3d).

### Isomin is able to form filaments *in vitro*

**Figure 4:**
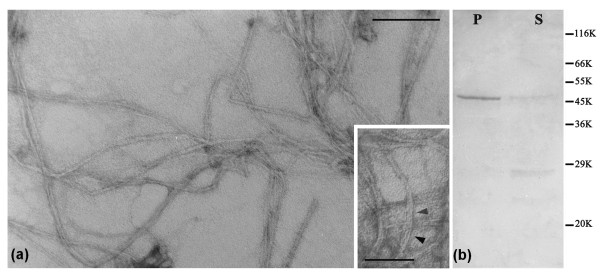
**Isomin in vitro reassembly**. (a) Negative staining of in vitro-reassembled isomin filament. Bar = 200 nm. The inset shows at a greater magnification two loosely packed filaments, revealing thinner protofilaments (arrowheads); bar = 100 nm. (b) Electrophoretic analysis on a 12% SDS-polyacrylamide gel of the sedimented (P) and supernatant (S) fraction after centrifugation of *in-vitro *reassembled isomin filaments. Migration of molecular weight reference proteins is indicated on the right.

A hallmark feature of IF proteins is their capability to renature after solubilization by urea treatment and to undergo a spontaneous re-assembly process in appropriate conditions. Thus, we preliminarily tested the capability of recombinant isomin to assemble *in vitro *into filaments, using the standard re-assembly conditions described for other IF proteins [24]. Following this protocol, we observed the formation of morphologically distinct filaments (Figure 4a); parallel electrophoretic analysis showed them to consist essentially of the 40 kDa isomin band (Figure 4b). Most reconstituted filaments were about 9-12 nm in diameter but some unwoven filaments also occurred, revealing the presence of thinner, 2-4 nm protofilaments (see arrowheads in Figure 4a, inset). The *in-vitro *formation of such 'loose' filaments could result from suboptimal reassembly conditions. The requirement for peculiar reassembly conditions might be related to the unusual structure of the isomin molecule, which is characterized by an extremely short head domain and by a modified coil 1a segment. In addition, we do not know whether, *in vivo*, isomin forms homopolimers or is, instead, part of a heteropolimeric IF system, requiring a partner to correctly assemble into stable filament.

Though further studies are required to address these points, our first insight into the reassembly properties of isomin clearly indicates its capability to assemble into filaments.

### Phylogenetic analysis

**Figure 5:**
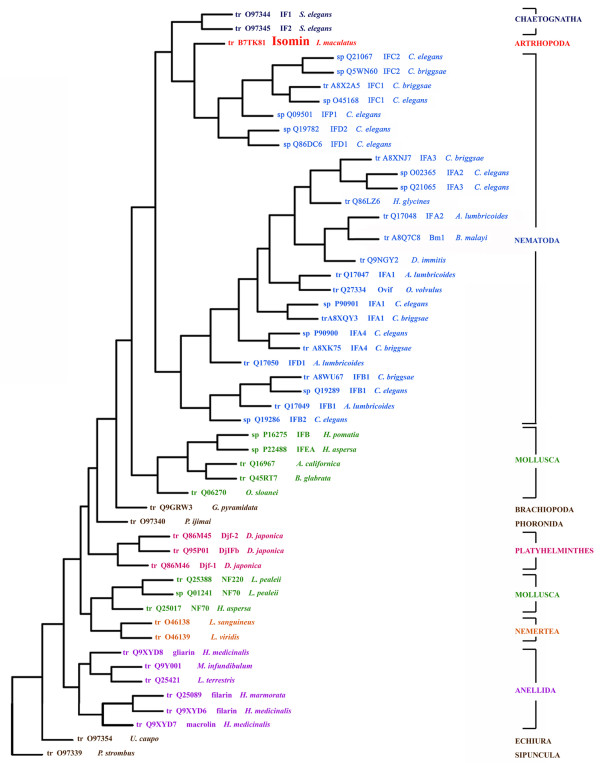
**Distance analysis of isomin and protostome cytoplasmic intermediate filament (IF) proteins**. Reconstruction of the phylogenetic tree was performed using the neighbour-joining method of the PHYLIP software package. GenBank accession numbers, referred to the trmbl (tr) and swiss-prot (sp) databank, respectively, are followed by species names. Different phyla are represented by diverse colours. Note: molluscan IF proteins cluster into two groups, corresponding to neuronal and non neuronal IF proteins. Only cytoplasmic complete IF sequences from protostome species have been included in this analysis.

When isomin is compared with the cytoplasmic IF sequences available in databank from other protostome phyla, it appears to cluster with those nematode IF proteins forming the IFC, IFD and IFP classes (Figure 5). Remarkably, the *C. elegans *IF proteins that cluster together with isomin are all constituents of the intestinal apical terminal web [17] and, like isomin, proteins IFC-1 and IFC-2 are characterized by a shorter tail domain for the lack of the lamin-homology segment that occurs in the tail of all other protostome cytoplasmic IFs [6,20].

## Discussion

### Isomin is a true but peculiar intermediate filament protein

All the members of the IF protein family share typical biochemical properties, the same molecular organization, peculiar sequence aspects and the capability to reassemble spontaneously *in vitro*, without requiring cofactors. Our results indicate that isomin possesses all the IF distinctive features.

First, it forms cytoskeletal filaments that are part of the detergent-resistant fraction of the cell. Second, it follows the consensus tripartite molecular architecture based on a central coiled coil domain flanked by non-helical terminal extensions. Third, it possesses all the signature sequences that characterize IF proteins: the helix initiation and termination motifs and the conserved discontinuity in the heptade phase in coil 2. Fourth, it is able to spontaneously self-assemble into filaments *in vitro*. Thus, the accumulated evidence clearly establishes isomin as a new member of the IF protein family.

However, some very peculiar aspects do occur in the first coiled coil segment of the isomin molecule, making this protein a very unique member of the IF family. This part of the molecule appears to deviate from the consensus organization of coil 1a subdomain. In isomin, this region retains the capability to form a coiled coil structure only over its aminoterminal part, which comprises the helix initiation motif, and only the presence of this conserved sequence clearly indicates that it is related to coil 1a of other IF proteins. Thus, a strong sequence drift has influenced coil 1a in isomin which appears, however, to be still compatible with the filament assembly process. It is interesting to note that the glycine and serine-rich sequence following the residual coiled coil segment is predicted to possess a high flexibility. This is a structural feature that, in most IF proteins, characterizes the L1 spacer and which is crucial for the correct IF assembly [4]. Thus, the occurrence of the helix initiation motif followed by a flexible region might still provide the isomin molecule with the essential structural features required for filament assembly.

It is also worth noting that, in IF proteins, coil 1a is thought to possess properties distinct from the other rod subdomains [25]. The possibility that it might form a dynamic structure able to switch between a two-stranded coiled-coil and an open conformation consisting of two separate α-helical strands has been proposed for vertebrate vimentin [26]. It has also been suggested that the capability of coil 1a to undergo a structural rearrangement might be important during the elongation phase of vimentin IF assembly which occurs by the longitudinal annealing of short, but already full-width, unit-length filaments (ULFs). The assembly of the long-rod invertebrate cytoplasmic IFs follows a different route, which does not imply the formation of ULFs [27]. Rather, tetramers anneal longitudinally to form long protofilaments which then associate laterally to yield the mature filament; this process is essentially mediated by the antiparallel association of coil 2 domains [27]. It is possible that this route of IF assembly is more compatible with the deviations from the consensus structure that have occurred in isomin coil 1a.

Isomin exhibits other unusual features besides the striking divergence observed in coil 1a region. First, it possesses very short end domains: it is tempting to relate the notable reduction of the head region - which is only 13 residues long - to the divergence of the aminoterminal part of the rod domain discussed above. Second, the tail domain is characterized by both an unusual aminoacid composition and the absence of the so-called lamin-homology domain which is a feature shared by all protostome IF proteins, with only a very few exceptions (see below). Third, the discontinuity in the heptad repeat, that is observed in all IF proteins at a conserved position of coil 2, consists in isomin of the insertion of two rather than of four positions. Fourth, coil 1b and coil 2 subdomains are characterized by overall basic properties which is quite an unusual feature among IF rod domains that more commonly show acidic charge features.

Thus isomin, while showing the common IF molecular hallmarks, exhibits a considerable divergence from the IF consensus structure.

### Evolutionary considerations

Isomin is the first cytoplasmic intermediate filament that, so far, has been found to be expressed in an arthropod species. Until now, arthropods have been thought not to express cytoplasmic intermediate filaments on the basis of both electron microscopy and molecular cloning [6,9,10]. However, these studies referred to a limited number of species. *I. maculatus *belongs to collembolans, a group of basal hexapods that has been recently positioned close to the branching of the arthropod phylogenetic tree, among Pancrustacea [28]. So, with respect to the other species analysed, collembolans might be more likely to have retained characters which occurred in the common ancestor but that have been subsequently lost during arthropod evolution.

Both the organization of the cytoskeletal array it assembles *in vivo *and some of its molecular aspects relate isomin to a group of IF proteins that are expressed in the intestinal epithelium of nematodes. The filamentous terminal web found in *I. maculatus *is ultrastructurally very similar to that described in some nematodes (compare our results with Figure 2 in [16]). Notably, the occurrence of such a peculiar array of IFs in the sub-microvillar region of the intestinal epithelium has not, so far, been detected in any other invertebrate group, although most phyla are likely to express IF proteins in their internal epithelia [9].

At the molecular level, phylogenetic analysis relates isomin to a group of nematode proteins - the members of the Intermediate Filament protein C, D and P (IFC, IFD and IFP) subfamilies. Remarkably, these proteins are all components of the terminal web in *C. elegans *intestine [17]. In particular, some of the unusual structural features that are exhibited by the isomin molecule (the absence of the lamin-homology segment as well as the occurrence in the tail of motifs rich in glycine and serine residues) are also present in the worm IFC-1 and IFC-2 proteins [6,20]. Moreover, IFC-2 has been reported to possess in coil 2 the same type of unusual structural discontinuity we have found in isomin [20]. These findings relate isomin mostly to the IFC nematode proteins and it is interesting to note that the latter are divergent proteins within the *C. elegans *IF family [6,20].

It is important to stress that the observed relatedness between isomin and nematode IFs is consistent with, and provides further support to, the recently established phylogenetic relationship between arthropods and nematodes. Nematodes have been proposed to be a sister group of arthropods within the Ecdisozoa clade on the basis of morphological, developmental and molecular characters [29]. In this context, the expression of an IF terminal web in the midgut of *Isotomurus *species appears to be evolutionarily related to the original intestine organization present in the ancestor common to arthropods and nematodes.

In *C. elegans*, IFs have been proposed to provide the intestinal wall with a high degree of mechanical and osmotic resilience. This view is mainly based on the observation that down-regulation of the IFC-2 protein induces multiple bubble-shaped invaginations of the lumen into intestinal cells, suggesting the occurrence of an increased epithelial fragility [30]. At the same time, the integrity of the terminal web is not required for the establishment and maintenance of the epithelial polarity, since the general organization of the apical domain and the junctional integrity are not affected. As to the other components of the terminal web, while the loss of a single protein, other than IFC-2, does not induce major defects on intestine organization [30], a pronounced defective phenotype is observed after simultaneous down-regulation of two or three IF proteins [31] - a fact that has been interpreted in light of the redundancy of IF proteins in nematodes.

Thus, the available evidence points to a central role for IFs in nematode intestine. This organ is subjected to a substantial mechanical stress from all directions, due both to the transport of nutrients and to the peculiar body plan organization and motility of these organisms, which are dominated by the high hydrostatic pressure present within their body cavity. The terminal web possesses peculiar mechanical properties able to provide the apical region of the cell with enhanced stability, as it is also indicated by the possibility to dissect, from various nematode species, the so-called endotube, a single and mechanically resistant unit which consists of both microvilli and the apical layer of the epithelium, including the web [16].

Isomin may have a similar stabilizing role in *Isotomurus *midgut epithelium. Notably, the terminal web is by far the most stable cellular component also in the intestine of this organism, since, at molt, it persists in the lumen as a compact filamentous network even after cell lysis (unpublished results).

However, during arthropod evolution, the accomplishment of a protective cuticle, in addition to maintaining a constant internal environment, has relieved most organs - and also the intestine - from external mechanical stress. In the light of this, the peculiarities observed in the molecular organization of isomin may either serve specific functions or, on the contrary, may be indicative of an increased rate of molecular drift which is, in turn, related to the development of the cuticle and of the new arthropod body plan. Thus, the reinforcing IF-based intestinal cytoskeleton - which is not essential for the establishment of epithelial polarity and function - is no longer subject to functional constraint and the onset of structural variations in its molecular components is more likely to occur. If the latter was the case, isomin should then be considered as a sort of molecular remnant leading to the loss of IF proteins during arthropod evolution.

## Conclusions

Our results provide the first characterization of an IF protein from an arthropod species and set a new basis for the analysis of IF protein evolution during arthropod phylogeny. In the light of this new information, the statement that the arthropod phylum lacks cytoplasmic IFs is no longer tenable. The unusual structure of the isomin protein and the strong molecular drift that has influenced the original coil 1a segment suggest the possibility that during arthropod evolution IF proteins might have subsequently accumulated further molecular modifications, eventually leading to the loss of IFs or to the expression of IF-related proteins with modified organizations that fail to assemble in canonical filaments, and may, therefore, be difficult to identify. A very limited number of species from this huge phylum has been analysed so far and further thorough molecular analyses on a more representative number of species will be necessary to address this important question.

## Methods

Specimens of the collembolan species *Isotomurus maculatus *(Arthropoda, Schäffer 1986) were collected in the neighbourhood of Siena and dissected in phosphate buffered saline (PBS; 171 mM NaCl, 6 mM Na phosphate, 3 mM KCl, 2 mM EDTA, 2 mM EGTA, pH 7.4). Midguts were then processed for electron microscopy, protein analysis, RNA extraction and immunomicroscopy as described below.

### Cytoskeletal preparations

Midgut cytoskeletal insoluble fractions were obtained following the procedure described by Pruss *et al*. [32]. Briefly, 300-500 midguts were homogenized in a volume of 1.5 mL of PBS added with a cocktail of protease inhibitors (P2714, Sigma, NY, USA) and centrifuged at 10,000 *g *for 10 min at 4°C. The sediment was then extracted in 1.5 mL of PBS containing 0.6 M KCl and 0.5% Triton X-100 and centrifuged as above. The resulting pellet was washed twice in PBS and then either fixed for electron microscopy analysis or denatured for successive sodium dodecyl sulphate (SDS)-acrylamide gel electrophoresis.

### Gel electrophoresis and immunoblot

Electrophoretic analysis of cytoskeletal fractions was carried out on 8% or 12% SDS-polyacrylamide gels according to the method used by Laemmli [33]. Gels were either stained using standard Coomassie blue staining procedures or transferred onto nitrocellulose for subsequent immunoblot analysis.

Electrophoretic transfer of proteins onto nitrocellulose was performed as described by Towbin *et al*. [34], using a modified transfer buffer, consisting of 50 mM Tris, 38 mM glycine and 5% methanol. Transferred protein bands were immunostained using the affinity-purified anti-isomin polyclonal antibody (see below) and a peroxidase-labelled anti-rabbit secondary antibody (Cappel, PA, USA). Blots were then processed for ECL detection (GE Healthcare, NJ, USA), with exposure times from 5 s to 5 min.

### Protein analysis by mass spectrometry (LC-ESI-MS/MS)

Analysis by mass spectrometry (LC-ESI-MS/MS) of the main tryptic fragments obtained by digestion of the isomin band was performed by the Swiss-2D Service (Geneva, Switzerland); the obtained peptide aminoacid sequences are listed in Additional File 1, Table S1.

### RNA preparation and RT-PCR

Total RNA was isolated from adults of *I. maculatus *frozen in liquid nitrogen and homogenized using a Polytron homogenizer (Kinematica AG, Littau, Switzerland) according to the method described by Chomczynski and Sacchi [35]. RNAs (1 μg) were converted to cDNAs with an oligo(dT)_18 _primer and SuperScript™ II Reverse Transcriptase (Invitrogen, CA, USA) according to manufacturer's instructions. Forward and reverse degenerate primers were designed on the basis of three amino acid sequences obtained by mass spectrometry: (B1-B2) YEAEAAK, (C1-C2) ESTGDAE and (D1-D2) FGHADQEK. Their sequences are listed in Additional File 1, Table S1. A partial 360 bp fragment of the isomin protein was amplified by RT-PCR using the combination B2/D1 of degenerate primers. Reactions were performed using Expand High Fidelity PCR System (Roche, Basel, Switzerland) under the following conditions: 95°C for 10 min; cycles 1-5: 94°C for 1 min, 35°C for 1 min, 72°C for 1 min; cycles 6-46: 94°C for 1 min, 54°C for 1 min, 72°C for 1 min and 72°C for 10 min. PCR products were purified using Wizard^® ^SV Gel and PCR Clean-Up System (Promega, WI, USA), cloned into a TA cloning vector (Invitrogen) and sequenced using a CEQ 8000XL automated DNA Analysis System (Beckman Coulter, CA, USA) on both strands. The 360 bp deduced amino acid sequence showed the complete sequences used to design B2 and D1 degenerate primers plus an additional one corresponding to the mass spectrometry fragment 5 (Additional File 1, Table S1).

### Cloning of full length cDNA by rapid amplification of the complementary DNA ends (RACE)

In order to obtain the full-length cDNA for isomin, a 5' and 3' RACE was performed on *I. maculatus *total RNA using a GeneRacer™ Kit (Invitrogen) and following manufacturer's instructions. Isomin specific primers - D1_FEcoRI, Iso112_for (3' nested primer; Additional File 1, Table S1) used in 3' amplification, B2_RBamHI and iso577_rev (5' nested primer; Additional File 1, Table S1) used in 5' amplification - were designed based on the partial 360 bp sequence mentioned above. Both nested PCR reactions gave a single product of ≈1200 bp and ≈ 500 bp, respectively. The 5' and 3' RACE products were purified, cloned and sequenced as described above. The complete isomin cDNA sequence has been deposited in GenBank under accession number FJ264504.

### Sequence analysis

Homology searches for nucleotide and amino acid sequences were performed using the BLAST suite of programs (http://blast.ncbi.nlm.nih.gov/Blast.cgi). Programs employed for the prediction of molecular domains implicated in coiled coils formation were Coils (http://www.ch.embnet.org/software/COILS_form.html) and Marcoil (http://bioinf.wehi.edu.au/folders/mauro/Marcoil/index.html); sequence alignments were obtained using ClustalW (http://www.ebi.ac.uk/Tools/clustalw2/index.html). Prediction of phosphorylated sites and of sumoylated residues was performed using NetPhos 2.0 (http://www.cbs.dtu.dk/services/NetPhos/) and SUMOsp 2.0 (http://sumosp.biocuckoo.org/prediction.php), respectively. Distance analysis of protostome cytoplasmic IF sequences was carried out using the Protdist and Neighbor programs of the Phylip package (JTT matrix, 100 bootstrap replicates) available at the Pasteur Institute web server (http://mobyle.pasteur.fr/cgi-bin/portal.py).

### Bacterial expression of recombinant protein

For the preparation of the glutathione S-transferase (GST) fusion constructs, either a portion (360 bp, coding the protein region from Phe_11 _to Lys_131_) or the whole isomin protein coding region (1236 bp) were amplified by PCR. Oligonucleotide pairs containing sites for in-frame directional cloning in the pGEX-6P-2 vector (GE Healthcare, NJ, USA) were: D1_FBamHI/B2_RSmaI for the 360 bp fragment and isoml_BamHI/isoml_SmaI for the 1236 bp one (Additional File 1, Table S1). PCR cycling, for the short fragment amplification, included an initial denaturation at 94°C for 5 min followed by 40 cycles of 94°C for 1 min, 52°C for 1 min, 72°C for 1 min and a final extension step at 72°C for 10 min. The 1236 bp region was amplified under the same PCR conditions but with an annealing temperature of 56°C. The PCR products were cloned into the BamHI and SmaI sites of the bacterial expression vector pGEX-6P-2 (GE Healthcare, NJ, USA) in frame with the upstream gene GST. Expression of the fusion protein was induced in *E. coli *by addition of Isopropyl-β-D-thiogalactopyranoside (IPTG; see below).

### Purification of the fusion protein and production of specific antibodies

Recombinant bacterial strains expressing the fusion protein - either the whole isomin molecule or its 120 aa fragment from Phe_11 _to Lys_131 _(see Additional File 2, Figure S1) - were grown at 37°C in 400 mL of Luria-Bertani (LB) medium added with 50 μg/mL of ampicillin, until an OD_600 _of about 0.6-0.8 is achieved. After the addition of IPTG to a final concentration of 0.1 mM, the culture was incubated at 37°C for 2 h under shaking. Cells were harvested by centrifugation at 3500 *g *for 10 min, resuspended by vortexing in 25 mL of B-PER™ reagent (Pierce) and shaken at room temperature for 10 min; soluble proteins were separated by insoluble material by centrifugation at 27000 *g *for 15 min.

Most part of the fusion protein was contained in inclusion bodies. Only in the case of the 120 aa fragment it was possible to recover an amount of soluble protein sufficient for the subsequent purification by affinity chromatography on Gluthatione Sepharose 4B (GE Healthcare, NJ, USA). This procedure was carried out according to manufacturer's instructions. Briefly, the recovered soluble fraction was added with 1 mL of a 50% slurry of resin and incubated for 1 h with gentle agitation. Resin was then collected by centrifugation at 500 *g *for 5 min and washed twice in PBS. Protein bound to the resin was eluted with 10 mM glutathione in 50 mM Tris-HCl pH 8.0, dialyzed against 0.9% NaCl and used for rabbit immunization following standard procedures. The resulting polyclonal antibodies were blot-affinity purified according to Tang (36), using nitrocellulose membrane fragments containing the isomin 40 kDa protein band.

### Isomin *in vitro *reassembly

Recombinant isomin was essentially recovered in the insoluble inclusion bodies. For their purification, the insoluble pelleted material obtained from a 50 mL-culture after cell lysis was resuspended by vortexing in 5 mL B-PER™, added with 200 μg/mL lysozime and incubated at room temperature for 5 min. The suspension was then added with 15 mL of B-PER™ diluted 1:10 in 20 mM Tris pH 7.5 and centrifuged for 15 min at 27000 *g*. Pelleted inclusion bodies were washed twice in B-PER™ diluted 1:10 in 20 mM Tris pH 7.5, then twice in 2% TRITON 10 mM EDTA. The final pellet essentially consisted of a band of about 66 kDa, corresponding to the recombinant protein (Additional File 5, Figure S4A). For cleavage of the GST moiety, pelleted inclusion bodies were thoroughly resuspended at about 75 μg/mL in a buffer consisting of 50 mM Tris-HCl, 150 mM NaCl, 1 mM EDTA, 1 mM DTT, pH 7.0, added with 10 U of PreScission Protease (GE Healthcare, NJ, USA) and incubated at 5°C for 20 h. After centrifugation at 8000 *g *for 20 min, both supernatant and sediment fractions were analysed by SDS-page (Additional File 5, Figure S4B); most part of the recombinant protein was shown to be cleaved and still to be contained in the insoluble fraction, while the GST protein was recovered in the soluble fraction.

The insoluble pellet recovered after cleavage was dissolved in 10 mM Tris, 9.5 M urea, pH 7.0 and incubated for 1 h at room temperature. After centrifugation for 1 h at 150000 *g*, analysis by SDS-page of the soluble and insoluble fractions revealed that urea treatment solubilised about 50% of the isomin protein (Additional File 5, Figure S4C). As described by Herrmann *et al*. [24], urea-solubilized protein was then dialyzed against a series of solutions (1 h each) containing decreasing concentrations (8 M, 6 M, 4 M, 2 M) of urea dissolved in 5 mM Tris, 0.1 mM EGTA, 1 mM EDTA, 1 mM DTT, pH8.4 and, finally, against the same buffer containing no urea. After dialysis, the sample was added with an appropriate volume of 10× reassembly buffer (0.2 M Tris, 0.5 M NaCl, pH 7.0), incubated for 1 h at room temperature and then centrifuged at 150000 *g *for 1 h. Sediment and supernatant were analysed by both SDS-page and negative staining.

### Immunomicroscopy

For immunofluorescence microscopy, midguts were fixed in absolute ethanol for 1 h at 4°C and then paraffin-embedded. After rehydration, sections were treated with 1% NaBH_4_ for 30 min to quench autofluorescence and washed twice for 5 min with PBS. Successively, sections were incubated for 1 h in PBS containing 3% bovine serum albumine and 0.5% Tween-20, and for 2-4 h in the affinity-purified primary antibody. After two 10-min washes in PBS containing 0.1% Tween-20, sections were incubated for 1 h in the secondary antibody (fluorescein-conjugated anti-rabbit antibodies, Cappel), washed again in PBS-T and finally mounted in 90% glycerol.

### Electron microscopy

Whole midguts or midgut TRITON-resistant fractions were fixed with glutaraldehyde and tannic acid, post fixed with uranyl acetate and embedded in an Epon/Araldite mixture as previously described in [37]. Before observation by TEM, thin sections were routinely stained with uranyl acetate and lead citrate.

For post-embedding electron microscopy immunolocalization, whole midguts were fixed at 4°C for 2 h in 0.1 M phosphate buffer (PB) pH 7.2, containing 0.2% glutaraldehyde and 2% p-formaldehyde, rinsed overnight in PB, dehydrated at 4°C in a graded ethanol series and embedded in Lowicryl K4M as described in [38]. For immunogold staining, sections were saturated with 3% bovine serum albumin (BSA), 15% normal serum in PBS (2 h), treated with 20 mM glycine in PBS (20 min), and then incubated overnight at 4°C in the primary antibody diluted in PBS containing 0.2% BSA. After one 10-min wash with PBS containing 0.5% Tween 20 and five 10-min washes in PBS, sections were incubated for 1 h at room temperature in the secondary antibody (GAM IgG-G10, Biocell, Cardiff, Wales, UK). Grids were then washed 5 × 10 min in PBS and 5 × 10 min in distilled water, then counterstained as above reported. All the ultrathin sections were observed at a Philips CM10 EM operating at 80 kV.

## Abbreviations

BSA: bovine serum albumin; GST: glutathione S-transferase; IF: intermediate filament; IPTG: Isopropyl-β-D-thiogalactopyranoside; RT-PCR = reverse-transcriptase polymerase chain reaction; RACE: rapid amplification of cDNA ends; cDNA; complementary DNA; SDS: sodium dodecyl sulphate.

## Authors' contributions

CM conceived the experimental project and wrote the paper. CM and DC performed the biochemical and immunolocalization experiments. SC performed the molecular studies. CM and SC were responsible for molecular data analyses and interpretation. RD and PL performed the ultrastructural analyses. All the authors discussed, revised and approved the final manuscript.

## Supplementary Material

Additional file 1 **Table S1**. Mass spectrometry fragments and primers used in polymerase chain reaction. Click here for file

Additional file 2 **Figure S1**. Nucleotide and deduced aminoacid sequences of isomin complementary DNA. The aminoacid stretches corresponding to the sequences obtained by mass spectrometry analysis of tryptic fragments are underlined; the 360 bp fragment that has been initially obtained by reverse transciptase polymerase chain reaction is shaded. The start codon and the poly(A) signal are underlined. The stop codon is marked with an asterisk. Click here for file

Additional file 3 **Figure S2**. Heptad repeats along the three coiled coil domains of the isomin molecule. The (abcdefg) positions of consecutive heptads are reported above the isomin aminoacid sequence; coiled coil domains are highlighted in grey. The arrowhead indicates the position of the stutter. The heptad positions and the ends of the coiled coil domains were inferred by comparison of the secondary structure prediction results with the sequence alignment of isomin and other invertebrate intermediate filament proteins. Click here for file

Additional file 4 **Figure S3**. Predicted sites of posttranslational modification in the isomin molecule: isomin is predicted to be a phosphorylated and sumoylated protein. Phosphorylable serine and threonine residues are in red, the two predicted sumoylated lysine residues are in green. Arrowheads indicate the region of the molecule comprised between the helix initiation and the helix termination motifs; coil 1b and coil 2 are highlighted in pale blue. Click here for file

Additional file 5 **Figure S4**. Purification of recombinant isomin. (A) Purified inclusion bodies contain a band of about 66 K, corresponding to the fusion protein GST-isomin. (B) After treatment of inclusion bodies with the preScission protease, isomin still occurs in the insoluble fraction (P), while GST is solubilised (S). (C) Urea treatment results in the solubilization of about 50% isomin from inclusion bodies. Electrophoresis on a 12% SDS-polyacrylamide gel. Click here for file
