# Supplementary figures and images for: Isomin: a novel cytoplasmic intermediate filament protein from an arthropod species
Source: BMC Biol. 2011 Feb 28;9:17. doi: 10.1186/1741-7007-9-17 (PMC3065449; doi:10.1186/1741-7007-9-17)

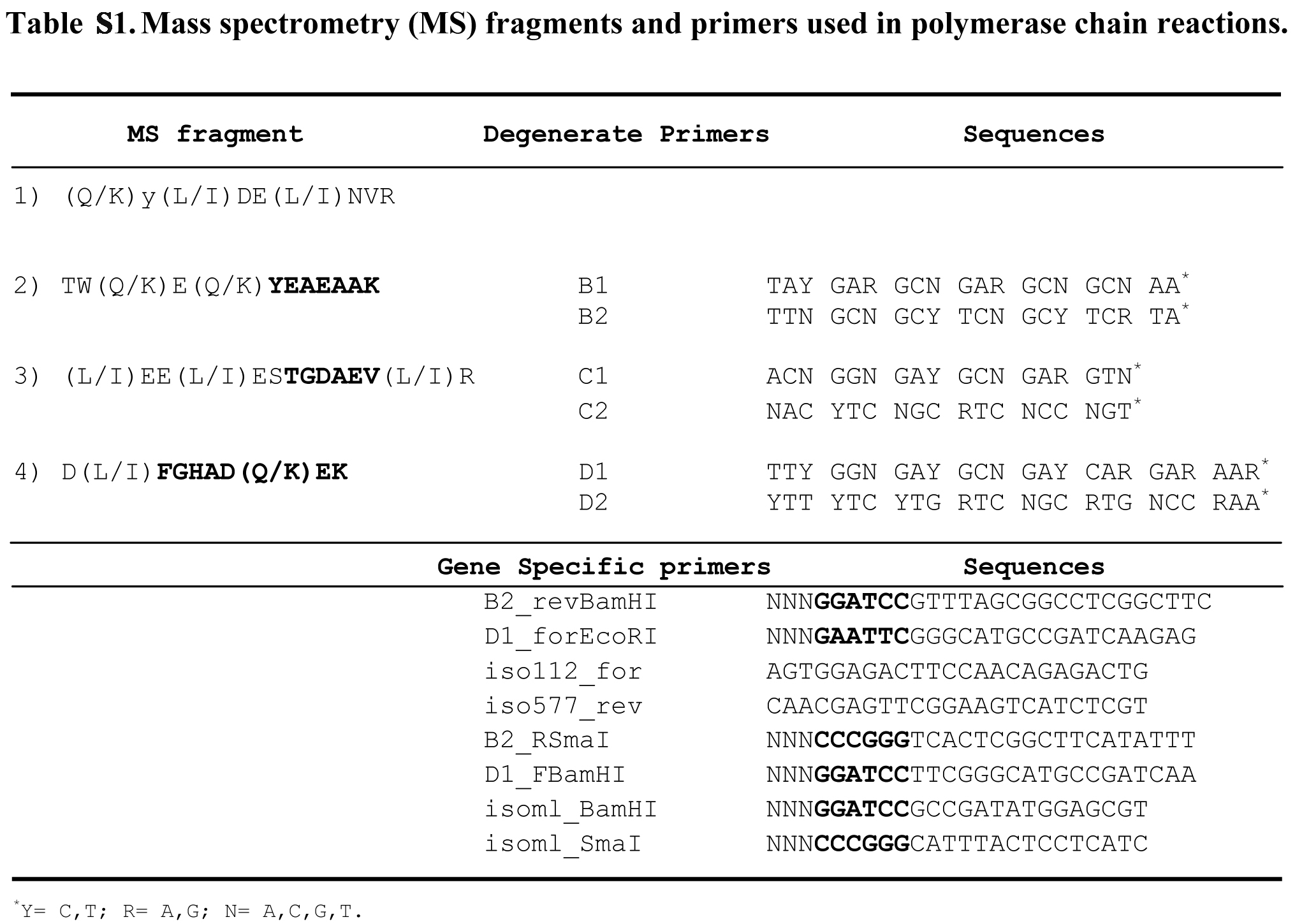

Supplement: Additional file 1 — Table S1. Mass spectrometry fragments and primers used in polymerase chain reaction. [file 1741-7007-9-17-S1.jpeg]

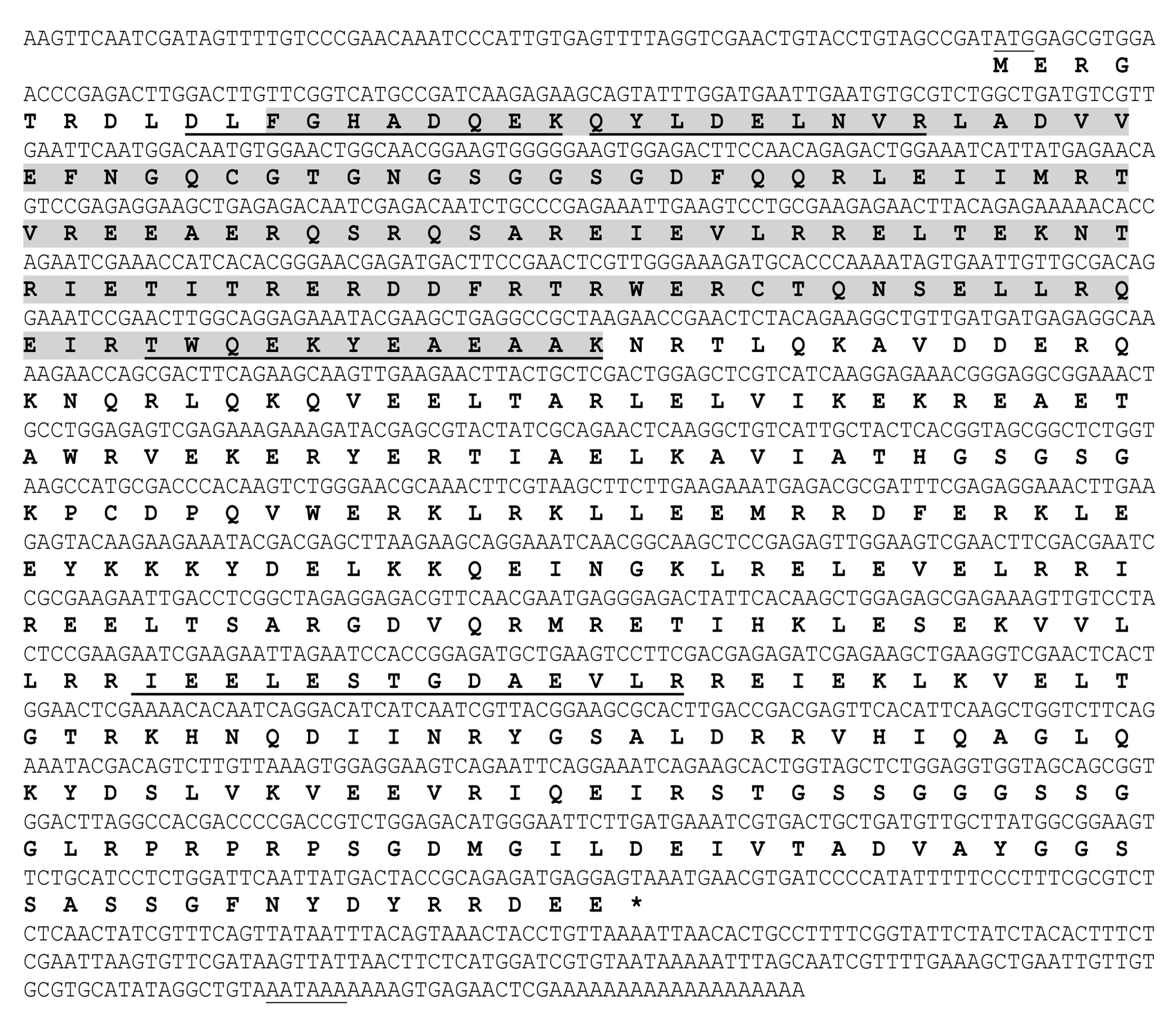

Supplement: Additional file 2 — Figure S1. Nucleotide and deduced aminoacid sequences of isomin complementary DNA. The aminoacid stretches corresponding to the sequences obtained by mass spectrometry analysis of tryptic fragments are underlined; the 360 bp fragment that has been initially obtained by reverse transciptase polymerase chain reaction is shaded. The start codon and the poly(A) signal are underlined. The stop codon is marked with an asterisk. [file 1741-7007-9-17-S2.jpeg]

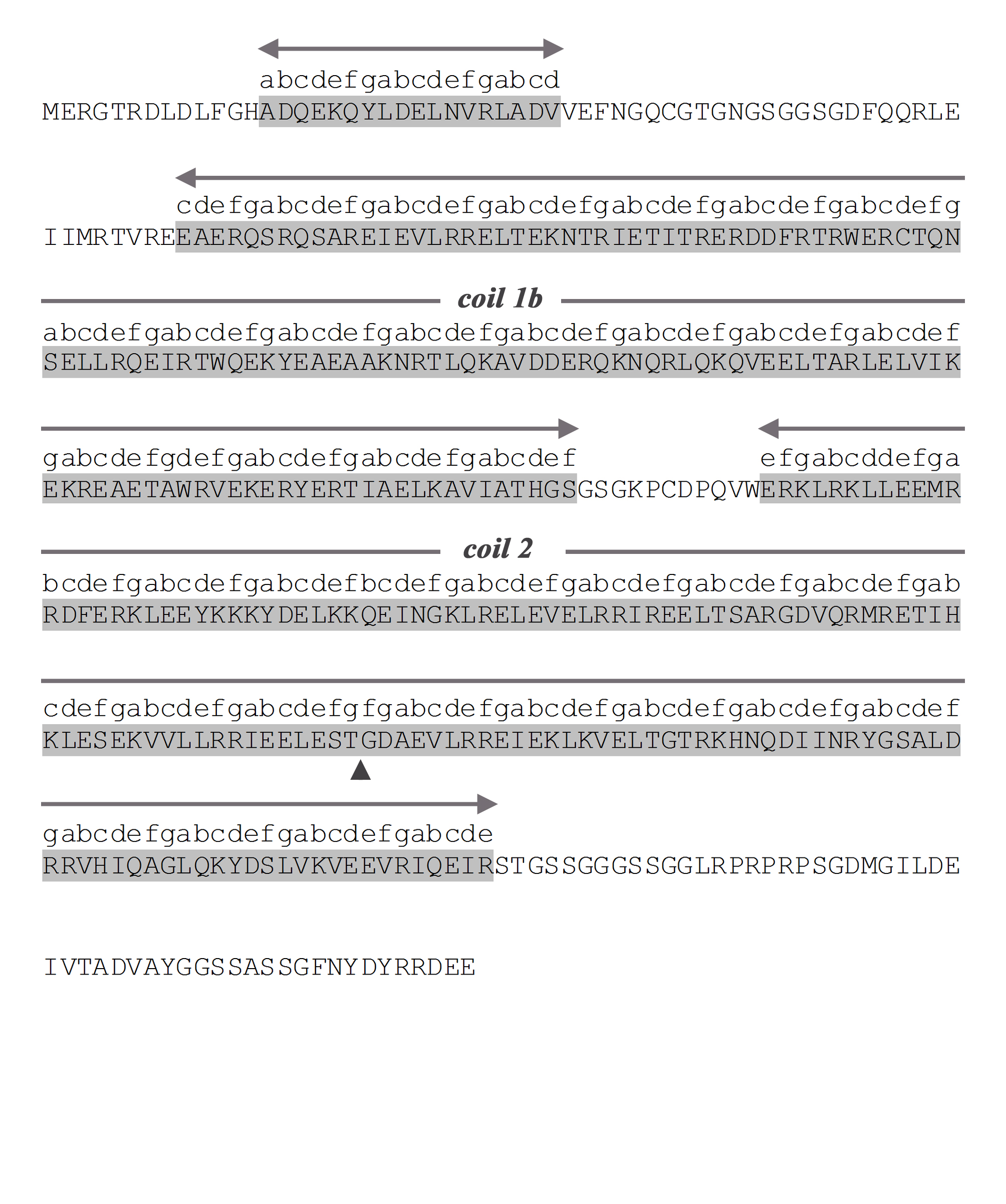

Supplement: Additional file 3 — Figure S2. Heptad repeats along the three coiled coil domains of the isomin molecule. The (abcdefg) positions of consecutive heptads are reported above the isomin aminoacid sequence; coiled coil domains are highlighted in grey. The arrowhead indicates the position of the stutter. The heptad positions and the ends of the coiled coil domains were inferred by comparison of the secondary structure prediction results with the sequence alignment of isomin and other invertebrate intermediate filament proteins. [file 1741-7007-9-17-S3.jpeg]

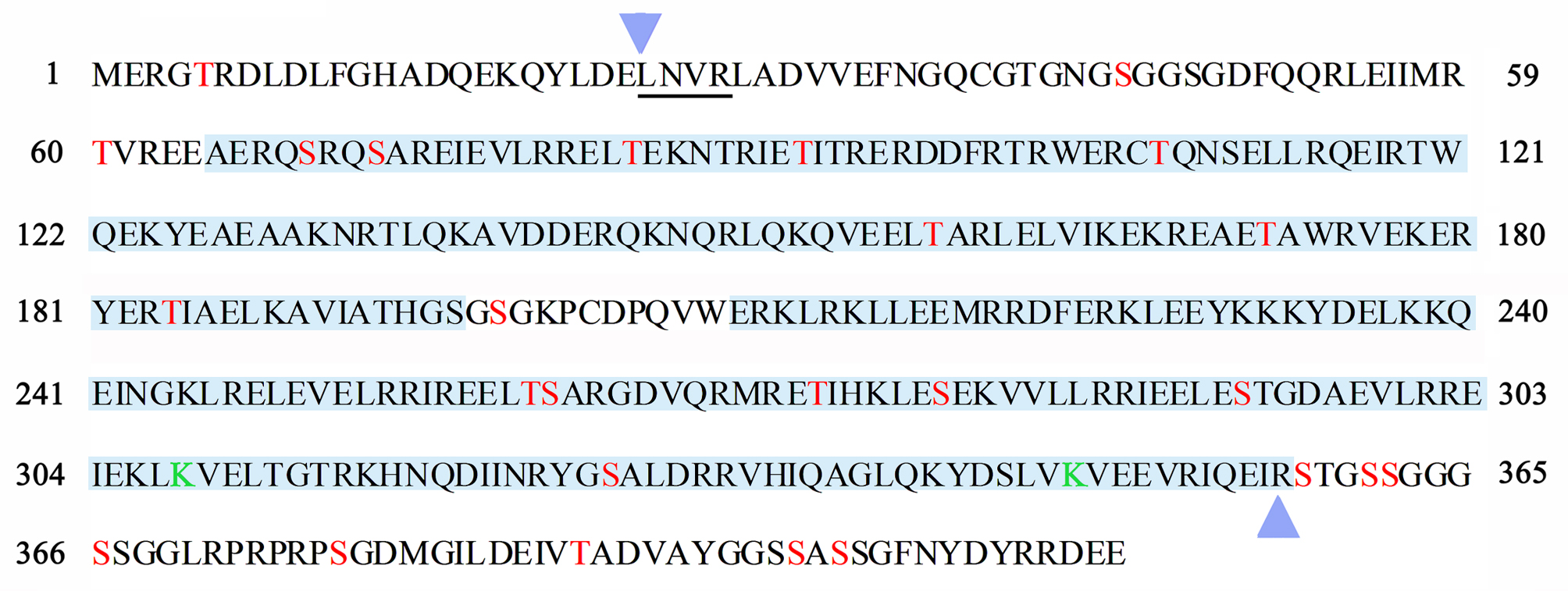

Supplement: Additional file 4 — Figure S3. Predicted sites of posttranslational modification in the isomin molecule: isomin is predicted to be a phosphorylated and sumoylated protein. Phosphorylable serine and threonine residues are in red, the two predicted sumoylated lysine residues are in green. Arrowheads indicate the region of the molecule comprised between the helix initiation and the helix termination motifs; coil 1b and coil 2 are highlighted in pale blue. [file 1741-7007-9-17-S4.jpeg]

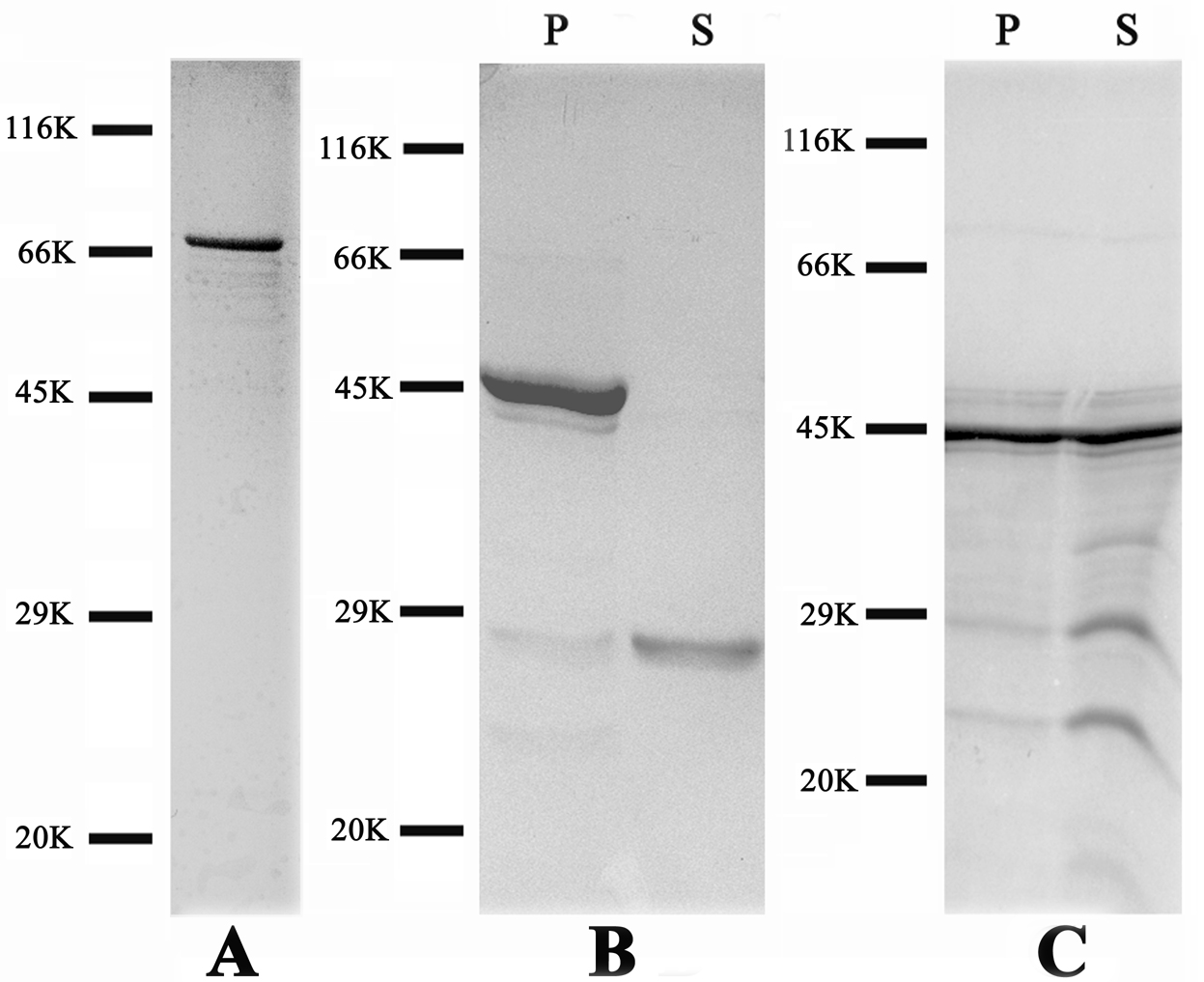

Supplement: Additional file 5 — Figure S4. Purification of recombinant isomin. (A) Purified inclusion bodies contain a band of about 66 K, corresponding to the fusion protein GST-isomin. (B) After treatment of inclusion bodies with the preScission protease, isomin still occurs in the insoluble fraction (P), while GST is solubilised (S). (C) Urea treatment results in the solubilization of about 50% isomin from inclusion bodies. Electrophoresis on a 12% SDS-polyacrylamide gel. [file 1741-7007-9-17-S5.jpeg]
